# Efficacy and safety of regenerative cell therapy for pulmonary arterial hypertension in animal models: a preclinical systematic review protocol

**DOI:** 10.1186/s13643-016-0265-x

**Published:** 2016-05-25

**Authors:** Colin M. Suen, Alex Zhai, Manoj M. Lalu, Christopher Welsh, Brendan M. Levac, Dean Fergusson, Lauralyn McIntyre, Duncan J. Stewart

**Affiliations:** Regenerative Medicine Program, The Ottawa Hospital Research Institute, 501 Smyth Rd, Ottawa, ON K1H8L6 Canada; Department of Cell and Molecular Medicine, University of Ottawa, Ottawa, Canada; Department of Anesthesiology, The Ottawa Hospital, The Ottawa Hospital Research Institute, Ottawa, Canada; Clinical Epidemiology Program, The Ottawa Hospital Research Institute, Ottawa, Canada; Department of Medicine, University of Ottawa, Ottawa, Canada; Department of Surgery, University of Ottawa, Ottawa, Canada; Department of Epidemiology and Community Medicine, University of Ottawa, Ottawa, Canada

**Keywords:** Pulmonary arterial hypertension, Preclinical, Systematic review, Risk of bias, Stem cells, Cell therapy, Endothelial progenitor cells, Mesenchymal stem cells, Animal models

## Abstract

**Background:**

Pulmonary arterial hypertension (PAH) is a rare disease (15 cases per million) that is characterized by widespread loss of the pulmonary microcirculation and elevated pulmonary vascular resistance leading to pathological right ventricular remodeling and ultimately right heart failure. Regenerative cell therapies (i.e., therapies involving cells with stem or progenitor-like properties) could potentially restore the effective lung microcirculation and provide a curative therapy for PAH. Preclinical evidence suggests that regenerative cell therapy using endothelial progenitor cells or mesenchymal stem cells may be beneficial in the treatment of PAH. These findings have led to the completion of a small number of human clinical trials, albeit with modest effect compared to animal studies. The objective of this systematic review is to compare the efficacy and safety of regenerative cell therapies in preclinical models of PAH as well as assess study quality to inform future clinical studies.

**Methods:**

We will include preclinical studies of PAH in which a regenerative cell type was administered and outcomes compared to a disease control. The primary outcome will be pulmonary hemodynamics as assessed by measurement of right ventricular systolic pressure and/or mean pulmonary arterial pressure. Secondary outcomes will include mortality, survival, right ventricular remodeling, pulmonary vascular resistance, cardiac output, cardiac index, pulmonary acceleration time, tricuspid annular systolic excursion, and right ventricular wall thickness. Electronic searches of MEDLINE and EMBASE databases will be constructed and reviewed by the Peer Review of Electronic Search Strategies (PRESS) process. Search results will be screened independently in duplicate. Data from eligible studies will be extracted, pooled, and analyzed using random effects models. Risk of bias will be assessed using the SYstematic Review Centre for Laboratory animal Experimentation (SYRCLE) risk of bias tool, and individual study reporting will be assessed according to an itemized checklist based on the Animal Research: Reporting of In vivo Experiments (ARRIVE) guidelines.

**Discussion:**

This systematic review will examine the efficacy and safety of regenerative cell therapy in preclinical models of PAH. As well, the literature will be assessed for study quality and risk of bias. The results will guide the design of future clinical trials and preclinical animal studies.

**Systematic review registration:**

CAMARADES (http://www.dcn.ed.ac.uk/camarades/SyRF/Protocols.htm).

**Electronic supplementary material:**

The online version of this article (doi:10.1186/s13643-016-0265-x) contains supplementary material, which is available to authorized users.

## Background

Pulmonary hypertension is a progressive disease that results from restricted blood flow through the pulmonary circulation and loss of effective pulmonary microvascular area. This leads to increased resistance in the pulmonary vasculature and eventually right heart failure [1]. Clinically, pulmonary arterial hypertension (PAH) is defined by an increase in mean pulmonary arterial pressure ≥25 mmHg at rest by right heart catheterization with a pulmonary capillary wedge pressure ≤15 mmHg. PAH, classified as World Health Organization (WHO) Group I pulmonary hypertension [1, 2], represents a group of diseases of various etiologies that are characterized by increased pulmonary vascular resistance due to pathology at the level of the precapillary arteriolar system. Although the mechanisms underlying the pathobiology of PAH are still unclear, it is thought that injury to the pulmonary endothelium leads to apoptosis which, in turn, triggers processes that reduce the effective lung vasculature, including widespread loss of functional microcirculation and obliterative remodeling of the small pulmonary arterioles due to the emergence of growth dysregulated vascular cells [3]. Ultimately, loss of lung microcirculation leads to progressive increase in pulmonary vascular resistance, right ventricular remodeling, and eventually right heart failure [4–6]. PAH is subdivided into subgroups based on etiology such as idiopathic, hereditary, drug- and toxin-induced, and PAH associated with other diseases such as connective tissue disease HIV, schistosomiasis, chronic hemolytic anemia, and congenital heart disease (Table 1).

**Table 1:** Clinical classification of WHO group 1 pulmonary hypertension

1. Pulmonary arterial hypertension (PAH)
1.1 Idiopathic PAH
1.2 Heritable PAH
1.2.1 BMPR2
1.2.2 ALK-1, ENG, SMAD9, CAV1, KCNK3
1.2.3 Unknown
1.3 Drug- and toxin-induced
1.4 Associated with:
1.4.1 Connective tissue disease
1.4.2 HIV infection
1.4.3 Portal hypertension
1.4.4 Congenital heart disease
1.4.5 Schistosomiasis
1′ Pulmonary veno-occlusive disease and/or pulmonary capillary hemangiomatosis
1″ Persistent pulmonary hypertension of the newborn (PPHN)

Adapted from Simonneau et al., JACC 2013 [1]

The current standard of care PAH-specific therapies consists largely of pharmacological vasodilator agents, such as phosphodiesterase-5 inhibitors, prostacyclin analogs, and endothelin antagonists. These have only modest effects on pulmonary hemodynamics, and prognosis remains poor despite introduction of a number of new therapies in the last 5 years [7]. The most recent estimate of 5-year survival of newly diagnosed PAH is 61.2 % [7]. Thus, the development of clinically effective strategies to restore normal pulmonary structure and function in established PAH are needed.

Recent understanding of the role of adult stem and progenitor cells in the maintenance of vascular homeostasis and repair of injury has stimulated interest in the potential for regenerative cell therapies for PAH. Most of the preclinical studies of cell therapy for PAH have used two cell types in particular, early-outgrowth endothelial progenitor cells (EPCs, also known as circulating angiogenic cells, myeloid angiogenic cells) and mesenchymal stromal cells (MSCs, also known as mesenchymal stem cells, adult stem cells) [8, 9]. EPCs and MSCs have been described as dynamic and responsive cells that can migrate to sites of vascular injury in several in vivo animal disease models [10], facilitating neovascularization and reducing inflammation [8]. As a treatment for PAH, preclinical studies involving these cell types have demonstrated efficacy in improving key pathological features of PAH such as cardiopulmonary hemodynamics, restoring the degenerated microvascular area, and reducing both right ventricular and pulmonary vascular remodeling [8]. A small number of clinical studies involving stem/progenitor cell therapy on PAH patients have been completed or are underway and show some promise in controlling the extent of the disease [3, 11]. However, to date, there has been no systematic synthesis of preclinical studies investigating stem cells therapy for the treatment of PAH.

In order to address this knowledge gap, we will conduct a preclinical systematic review of regenerative cell therapy for PAH. In contrast to narrative preclinical reviews of stem cells for PAH treatment, this systematic review will provide additional key evaluations. The meta-analysis will provide an estimation of the cumulative overall effect of stem cell treatment on pulmonary hemodynamics based on currently available data in preclinical studies. This systematic review will also attempt to evaluate the quality of currently available evidence based on risk of bias assessment and completeness of reporting, and the potential for publication bias. This data may impact the design of current preclinical testing of stem cell therapies and potentially influence the design of future clinical trials.

### Study questions

In preclinical studies of pulmonary arterial hypertension, do PAH animals receiving regenerative cell therapy exhibit improved pulmonary hemodynamics compared to PAH animals not receiving regenerative cell therapy and is regenerative cell therapy safe?

## Methods/design

### Protocol and registration

The protocol was developed by a research team of clinical (DS, AZ) and preclinical research scientists (CS, ML), experts in knowledge synthesis and translation (DF, LM). The protocol will follow the Preferred Reporting Items for Systematic Reviews and Meta-Analyses Protocols (PRISMA-P) checklist (Additional file 1: PRISMA checklist). The protocol is registered through the Collaborative Approach to Meta Analyses and Review of Animal Data from Experimental Studies (CAMARADES) website (http://www.camarades.info).

### Types of studies

This review will include controlled studies (randomized, pseudo-randomized and non-randomized) that evaluate the efficacy or safety of regenerative cell therapy for PAH.

### Types of preclinical animal models

We will include preclinical in vivo models of PAH that reproduce features of the pathophysiology associated and/or etiology of human PAH [1]. Specifically, the eligible animal models are the rodent monocrotaline (MCT), chronic hypoxia (CH), and SU5416 + chronic hypoxia (SU+CH) models. These are recognized as the most representative and most widely utilized methods of induction for modeling human PAH [12]. These models do not require invasive surgery or extensive manipulation that could be subject to technical variability and therefore provide a predictable disease phenotype. These models share in common the characteristic pathological features of human PAH: endothelial dysfunction, SMC proliferation, inflammation, and vascular narrowing and rarefaction resulting in pulmonary hypertension and right ventricular remodeling; however, only the SU+CH model reproduces the complex obliterative vascular lesions that are typical of human PAH [13]. Mouse models will not be considered for this systematic review as currently available models (chronic hypoxia) lack significantly elevated pulmonary pressures, right ventricular hypertrophy, and pulmonary arteriolar remodeling [12].

Animal models of PH secondary to other causes such as left heart disease, lung disease, or thromboembolism (WHO Groups 3-5) [4] will not be included. Genetically modified animals and will also be excluded. Since the purpose of this study to inform future decisions for designing clinical trials for adult populations, this study will exclude neonatal animal models of pulmonary hypertension.

### Types of interventions

The intervention group will include animals receiving any regenerative cell therapy (xenogeneic, syngeneic, or allogeneic cells from any tissue source). Experiments involving pretreatment of cells, co-treatment, and/or genetic manipulation (e.g., engineered to over- or under-express certain genes) will be classified as “cell modifications” for subgroup analysis. Studies using non-stem/progenitor cells (i.e., terminally differentiated cells such as mature endothelial cells, smooth muscle cells, or fibroblasts) as the therapeutic intervention will be excluded. Studies that do not include the administration of viable cells, for example, studies with only cell-free products derived from stem/progenitor cells such as conditioned media, will also be excluded.

### Types of control comparisons

The preclinical comparison group will include animals from studies that have had experimentally induced PAH but have not been administered a regenerative cell (vehicle control, control cell type, or no treatment).

### Timing of outcome measurements

Outcomes will be assessed at least 1 week after intervention to exclude the possibility of acute effects of cell administration.

### Preclinical primary endpoints

The current gold standard for the diagnosis and evaluation of clinical pulmonary hypertension is direct pulmonary hemodynamic measures by right heart catheterization. The primary endpoint will be direct measures of pulmonary hemodynamics (mean pulmonary arterial pressure, right ventricular systolic pressure) measured after administration of cells (Table 2). The primary outcome will be assessed at the end of the follow-up period.

**Table 2:** Quantitative measures of severity of pulmonary hypertension

Collection method	Parameter	Surrogate index for	Type of variable
Direct measurement	Right ventricular systolic pressure (RVSP)	Pulmonary hemodynamics [12, 30]	Continuous
	Mean pulmonary arterial pressure (mPAP)	Pulmonary hemodynamics [12, 30]	Continuous
	Cardiac output, cardiac index	Cardiac function [12, 30]	Continuous (calculated)
	Pulmonary vascular resistance (PVR)	Pulmonary hemodynamics	Continuous (calculated)
Post-mortem tissue collection	Morphometric RV hypertrophy (RV/LV+S)	RV remodeling [12, 30]	Continuous
Echocardiography	Pulmonary artery acceleration time (PAT)	Pulmonary hemodynamics [12, 30]	Continuous
	RV free wall thickness	RV remodeling [12, 30]	Continuous
	Tricuspid annular plane systolic excursion (TAPSE)	Cardiac function	Continuous
Ratio of deaths/total participants at experimental endpoint	Death	Mortality	Continuous
Survival time relative to induction	Survival	Survival	Continuous

### Preclinical secondary endpoints

We will collect data on all deaths and animal mortality. Right ventricular (RV) remodeling is a forerunner of right heart failure, which is characterized by decreased function and dilatation of the RV and strongly correlates with prognosis and survival in PH patients [14]. We will collect morphometric data on right ventricular remodeling expressed as the weight ratio of right ventricle/left ventricle + septum. Other measures of cardiac function and hemodynamics will also be collected to assess functional performance of the heart such as cardiac output and cardiac index (cardiac output/body weight) and pulmonary vascular resistance.

We will collect data from other noninvasive measures obtained by echocardiography to evaluate cardiac structure and pulmonary hemodynamics (Table 2). Mortality and survival will also be collected to evaluate the safety of the intervention.

### Electronic search methods for study identification

In consultation with the review team, electronic search strategies will be developed for each database by an experienced medical information specialist. Ovid MEDLINE®, Ovid MEDLINE® In-Process & Other Non-Indexed Citations, and EMBASE Classic + EMBASE will be searched. The strategy appended was used to search in MEDLINE (Additional file 2). The search strategy will be validated using the Peer Review of Electronic Search Strategies (PRESS) template by another information specialist [15].

Search strategies will use a combination of controlled vocabulary (for example stem cells, pulmonary hypertension) and keywords (for example, EPC, MSC, iPSC, HSC, PAH), and parsing will be formatted accordingly to each database. We will use modified animal filters from previously published methods [16, 17] validated for PubMed/MEDLINE and EMBASE. There will be no date restrictions on any of the searches. In addition, a manual review of the bibliographies of selected articles and relevant reviews will be performed. Only articles in the English language will be included in the review.

### Study selection

Titles and abstracts of search results will be screened independently by two individuals. Full text of all potentially eligible studies will be reviewed based on our eligibility criteria. Disagreements between reviewers will be resolved by consensus or by a third member of the systematic review team (DJS). Reasons for exclusion of potentially eligible studies will be recorded in accordance with the Preferred Reporting Items for Systematic Reviews and Meta-Analyses (PRISMA) guidelines developed for proper reporting of clinical systematic reviews [18].

### Data collection and process and data items

Data will be extracted independently by two individuals into standardized, electronic pilot-tested forms using DistillerSR software (https://distillercer.com). Specific data elements collected for this review are listed in Table 2.

### Assessment of risk of bias

Risk of bias will be assessed independently in duplicate for each included study using the Systematic Review Centre for Laboratory animal Experimentation (SYRCLE) risk of bias tool. The SYRCLE tool was adapted from the Cochrane Risk of Bias Tool to assess the methodological quality using criteria specific to animal studies (Table 3). Items in this tool include assessments for selection bias (sequence generation, baseline characteristics, allocation concealment), performance bias (random housing, blinding), detection bias (random outcome assessment, blinded outcome assessment), attrition bias (completeness of outcome data), and reporting bias. For each included study, each parameter for type of bias will be scored as low, high, or unclear risk of bias.

**Table 3:** SYRCLE risk of bias (RoB) tool

Item	Type of bias	Domain	Description of domain	Review authors’ judgment
1	Selection bias	Sequence generation	Describe the methods used, if any, to generate the allocation sequence in sufficient detail to allow an assessment whether it should produce comparable groups.	Was the allocation sequence adequately generated and applied?^a^
2	Selection bias	Baseline characteristics	Describe all the possible prognostic factors or animal characteristics, if any, that are compared in order to judge whether or not intervention and control groups were similar at the start of the experiment.	Were the groups similar at baseline or were they adjusted for confounders in the analysis?
3	Selection bias	Allocation concealment	Describe the method used to conceal the allocation sequence in sufficient detail to determine whether intervention allocations could have been foreseen before or during enrolment.	Was the allocation adequately concealed?^a^
4	Performance bias	Random housing	Describe all measures used, if any, to house the animals randomly within the animal room.	Were the animals randomly housed during the experiment?
5	Performance bias	Blinding	Describe all measures used, if any, to blind trial caregivers and researchers from knowing which intervention each animal received. Provide any information relating to whether the intended blinding was effective.	Were the caregivers and/or investigators blinded from knowledge which intervention each animal received during the experiment?
6	Detection bias	Random outcome assessment	Describe whether or not animals were selected at random for outcome assessment, and which methods to select the animals, if any, were used.	Were animals selected at random for outcome assessment?
7	Detection bias	Blinding	Describe all measures used, if any, to blind outcome assessors from knowing which intervention each animal received. Provide any information relating to whether the intended blinding was effective.	Was the outcome assessor blinded?
8	Attrition bias	Incomplete outcome data	Describe the completeness of outcome data for each main outcome, including attrition and exclusions from the analysis. State whether attrition and exclusions were reported, the numbers in each intervention group (compared with total randomized animals), reasons for attrition or exclusions, and any re-inclusions in analyses for the review.	Were incomplete outcome data adequately addressed?^a^
9	Reporting bias	Selective outcome reporting	State how selective outcome reporting was examined and what was found.	Are reports of the study free of selective outcome reporting?^a^
10	Other	Other sources of bias	State any important concerns about bias not covered by other domains in the tool.	Was the study apparently free of other problems that could result in high risk of bias?^a^

^a^Items in agreement with the items in the Cochrane Risk of Bias tool. Hooijmans et al*. BMC Medical Research Methodology* 2014 14:43 doi:10.1186/1471-2288-14-43

### Assessment of external validity

This review will record features that will facilitate judgments of external validity. External validity in preclinical research concerns the extent to which results can be generalized to different experimental measures, settings, and times [19]. These assessments aid in the ability to replicate experimental findings. External validity will be assessed by subgroup analysis of the primary outcome based on: age, gender, species and strain of animal, model of PAH, severity of PAH, use of cell preservation, tissue source of regenerative cell product, timing of cell administration, dose of cells, type of control, presence of cell modification, and number of participating study centers (Table 4). These data will help evaluate the effect of factors such as animal characteristics and regenerative cell preparation. As well, this will help identify optimal conditions for regenerative cell therapy to inform future clinical trials.

**Table 4:** Elements of external validity

Category	Specific items
Strain	e.g., Sprague-Dawley versus Fischer 344
Species	Rat, mouse, dog, pig, etc.
Age	Age in weeks, body weight
Gender	Male versus female versus mix of genders used
Model of PAH	MCT, CH, or SUHx
Severity of PAH	RVSP or mPAP in control group
Cell preservation	Fresh versus fresh from previously cryopreserved versus thawed cryopreserved product
Tissue source of regenerative cell product	Bone marrow versus peripheral blood versus cord blood
Timing of cell administration following induction of PAH	Early (<2 weeks) versus late (≥2 weeks) intervention
Follow-up duration	Primary outcome assessed at 1, 2, 3, and 4+ weeks after intervention
Route of cell administration	Intravenous versus intratracheal versus intraperitoneal versus intramuscular
Dose of cells	e.g., 1 × 10^6^ cells
Type of control	PBS versus normal saline versus fibroblasts versus heat-killed cells
Cell modification	Yes versus No
Number of participating study centers	Single versus multi-center

### Assessment of construct validity

Construct validity in preclinical research refers to the ability of a study to generalizable to a clinical scenario [20]. Threats to construct validity arise when experimental conditions (model, intervention, outcomes) are not representative of the clinical scenario.

We will assess construct validity based on a pre-defined checklist of factors aimed evaluate how closely the study resembles clinical PAH. The domains assessed will be animal subjects, outcome measures, modeling of disease, administration of intervention, and environment (Table 5). These will be recorded as yes/no answers.

**Table 5:** Elements of construct validity

Grouping	Recommendations from guidelines^a^	Specific application to PAH	Justification
Animal subjects	Model matches age of patients to clinical setting	Adult animals included	Typical onset of PAH occurs in adulthood [31, 32]
	Characterization of animal properties at baseline	RVSP or mPAP assessed at baseline	Confirmation that the model successfully establishes PAH
	Matching model to sex of patients in clinical setting	Both male and female animal included	Prevalence of PAH occurs in female versus males is 2:1 [31, 32]
Outcome measurements	Matching of measure to clinical outcome	Clinically relevant outcome reported (e.g., pulmonary hemodynamics, RV remodeling, cardiac function, mortality)	Clinically relevant outcomes may increase potential generalizability to clinical setting
		Long-term follow-up (>3 weeks post-intervention)	PAH is a chronic disease; long-term assessment increases reliability of findings
		Pulmonary hemodynamics assessed by direct catheterization	Right heart catheterization is the gold standard for diagnosing PAH
	Assessment of multiple manifestations of disease phenotype	Study reports ≥2 types of outcome measurements (pulmonary hemodynamics, RV remodeling, cardiac function, mortality, histopathological assessment of vascular lesions)	Efficacy in multiple manifestations of PAH may increase reliability of findings
Modeling of disease	Matching model to human manifestation	Criteria for PAH are met in disease control (mPAP >25 mmHg; RVSP >35 mmHg [12, 33])	PAH is induced successfully in the model
	Treatment response along mechanistic pathway	A molecular or cellular mechanism of treatment is measured and reported	Ensures therapy is producing a biological effect; ensures negative effects cannot be ascribed to a lack of biological activity
Administration of intervention	Matching timing of treatment delivery to clinical setting	Intervention is given after PAH is established (>2 weeks in animal models)	PAH usually present with symptoms before diagnosis
	Matching the duration/exposure of treatment to clinical setting	Evidence of cell persistence in any animal organ	Ensures presence of cells during course of treatment
	Matching model to co-interventions in clinical setting	Animals are on background medical therapy for PAH (e.g., Prostacyclins endothelin receptor antagonists, PDE5 inhibitors, calcium channel blockers)	PAH patients would be on conventional pharmacotherapy
Environment	Address confounders associated with setting, experimental setting	General anesthetic is not used during outcome measurements	Anesthetics may exert effects on cardiovascular system; patients undergo right heart catheterization under local anesthetic, echocardiography performed without anesthetics in patients

^a^Recommendations to reduce threats to construct validity were identified by [20]

### Description of reporting

We will apply the Animal Research: Reporting of In vivo Experiments (ARRIVE) guidelines to evaluate the quality of reporting in preclinical studies. The ARRIVE guidelines were developed by the National Centre for the Replacement, Refinement, and Reduction of Animals in Research (NC3Rs) to improve the transparent and comprehensive reporting of research methods and results for in vivo animal experiments [21].

### Data analysis

Continuous endpoints will be pooled using the ratio of weighted means using the inverse variance random effects method [22, 23]. Death will be analyzed as the mortality ratio of *n*_deaths_/*n*_total_ at defined endpoints and by mean survival in days. Statistical heterogeneity of included studies will be measured using the *I*^2^ test with 95 % confidence intervals [24]. An evaluation for the presence of publication bias will be conducted with funnel plots and Egger’s regression test [25].

Planned subgroup analyses will be examined on the primary endpoint of right heart catheterization hemodynamics (RVSP/mPAP). Heterogeneity will be analyzed for the following: PAH induction method, age, sex, strain, regenerative cell type, tissue origin of cells, timing of administration (<2 weeks post-induction, ≥2 weeks post-induction), follow-up period (0, 1, 2, 3, 4+ weeks post-intervention), route of delivery, cell dose, dose frequency, use of cell pretreatments and other enhancement strategies (e.g., gene transfection), and sample sizes of study.

## Discussion

The timing of this review is highly relevant, as clinical trials have been completed (NCT00257413, NCT00641836, NCT00469027) [11, 3 ,26]. So far, based on limited short-term data, the results of completed clinical trials have shown relatively modest benefits [11] compared to the effect sizes reported in some preclinical literature [27, 28]. Most of the evidence supporting regenerative cell therapy for PAH has been based on findings observed in prevention studies rather than treatment studies in established pulmonary hypertension [8], which may have contributed to the overestimation of the degree of their efficacy. Therefore, a thorough examination of the study design with attention to the timing of interventions and suitability of follow-up duration must be conducted to determine the validity of the available evidence and potential for clinical translation.

Systematic reviews should be valuable in stem cell research because of the high degree of heterogeneity of cell products used for therapy. As such, a secondary aim of this study will be to evaluate the relative efficacy between regenerative cell types. Although EPCs and MSCs have both been studied extensively in preclinical models, we anticipate that other cell products such as cells derived from embryonic stem cells or induced pluripotent stem cell may also have in vivo efficacy. Still, guidelines and/or criteria for what constitutes a particular regenerative cell type are ill-defined and when available, they are loosely defined based on an evolving understanding of stem cell biology [29]. In the absence of such standards, this review will address the level of transparency in reporting of pertinent information regarding stem cell isolation and culture techniques and quality control for each cell type, as well as the delivery and dosing methods.

We anticipate that this comprehensive synthesis will provide valuable information in translating stem cell therapy to the clinic. In addition, this review will have an immediate impact on preclinical research by highlighting knowledge gaps and areas to improve study design for future preclinical investigations of PAH.

## Abbreviations

ALK-1, activin receptor-like kinase 1; ARRIVE, Animal Research, Reporting of In vivo Experiments; BMPR2, bone morphogenetic receptor, type 2; CAMARADES, Collaborative Approach to Meta Analysis and Review of Animal Data from Experimental Studies; CAV1, caveolin 1; ENG, endoglin; EPC, endothelial progenitor cell; KCNK3, potassium channel subfamily K member 3; mPAP, mean pulmonary arterial pressure; MSC, mesenchymal stem cell, marrow stromal cell, or mesenchymal stromal cell; PAH, pulmonary arterial hypertension; PVR, pulmonary vascular resistance; RV, right ventricle; RVSP, right ventricular systolic pressure; SMAD9, mothers against decapentaplegic homolog 9; SYRCLE, Systematic Review Centre for Laboratory animal Experimentation.

## Additional files

Additional file 1: Preferred Reporting Items for Systematic review and Meta-Analysis Protocols (PRISMA-P) 2015 checklist: recommended items to address in a systematic review protocol*. (DOC 81 kb) Additional file 2: Appendix A1. MEDLINE Search Strategy. (DOCX 126 kb)
