# Supplementary material for: Efficacy and safety of regenerative cell therapy for pulmonary arterial hypertension in animal models: a preclinical systematic review protocol
Source: Syst Rev. 2016 May 25;5:89. doi: 10.1186/s13643-016-0265-x (PMC4880876; doi:10.1186/s13643-016-0265-x)
Supplement: Additional file 2: — Appendix A1. MEDLINE Search Strategy. (DOCX 126 kb) [file 13643_2016_265_MOESM2_ESM.docx]

**Appendix A1**

**MEDLINE Search Strategy**

Database: Embase Classic+Embase <1947 to 2014 July 02>, Ovid MEDLINE(R) In-Process & Other Non-Indexed Citations and Ovid MEDLINE(R) <1946 to Present> Search Strategy:

--------------------------------------------------------------------------------

1 Hypertension, Pulmonary/ (77671)

2 (pulmonary adj2 hypertens*).tw. (76144)

3 (PAH)tw. (36107)

4 or/1-3 (123104)

5 Monocrotaline/ (3008)

6 (monocrotalin* or MCT).tw. (10828)

7 5 or 6 (11229)

8 4 or 7 (131708)

9 exp Stem Cells/ (338939)

10 ((stem or progenitor* or mother or precursor*) adj1 cell$1).tw. (455508)

11 ("colony-forming" adj (cell$1 or unit or units)).tw. (38308)

12 (CFU or CFUs).tw. (71887)

13 ((embryoid or embroid or embryonic) adj2 (body or bodies or cell$1)).tw. (66276)

14 (angioblast* or hemangioblast* or haemangioblast*).tw. (6922)

15 myoblast$1.tw. (22839)

16 ((haemogenic or hemogenic) adj2 (endothelial or endothelium)).tw. (311)

17 ((osteoprogenitor adj cell$1) or OPC or OPCs).tw. (9125)

18 (side adj population adj cell$1).tw. (842)

19 (circulating angiogenic adj cell$1).tw. (299)

20 (CAC or CACs).tw. (8858)

21 IPS cell$1.tw. (3869)

22 hiPSC$1.tw. (1124)

23 common lymphoid progenitor$1.tw. (516)

24 (("Pre-B" or "Pro-B") adj1 (cell$1 or lymphocyte*)).tw. (8285)

25 (("B-cell" or "B-cells" or B-lymphcyte$1 or B-lymphoid$1) adj1 (immatur* or precursor* or progenitor* or transitional)).tw. (8221)

26 (("T-cell" or "T-cells" or T-lymphcyte$1 or T-lymphoid$1) adj1 (immatur* or precursor* or progenitor* or transitional)).tw. (5507)

27 (thymocyte* or T-lymphocyte*).tw. (209185)

28 ((mesenchymal adj3 (stem or stroma$1 or progenitor* or precursor*)) and cell$1).tw. (55069)

29 (MSC or MSCs or ADMSC or ADMSCs or BM-MSC or BM-MSCs or BMD-MSC or BMD-MSCs or BMDMSC or BMDMSCs).tw. (35017)

30 ((multipotent or multi-potent) adj3 (stroma$1 cell$1 or stem cell$1)).tw. (6514)

31 marrow stroma$1 cell$1.tw. (12298)

32 CFU-F$1.tw. (1384)

33 exp Mesoderm/cy [Cytology] (6036)

34 mesenchymal.tw. and exp Genetic Therapy/ (1740)

35 exp Bone Marrow Cells/ (237902)

36 (bone marrow adj3 cell$1).tw. (122869)

37 (BMD-EPC or BMD-EPCs or BMDEPC or BMDEPCs).tw. (23)

38 exp Stem Cell Transplantation/ (140672)

39 ((hematopoietic or hemato-poietic or HSC or HSCs) adj10 transplant*).tw. (45247)

40 ((autologous or auto-logous or auto) adj HCT).tw. (534)

41 autoHCT.tw. (61)

42 ((allogeneic or allo-geneic or homologous or homo-logous) adj HCT).tw. (1436)

43 ((PBSC or PBSCs) adj10 transplant*).tw. (2248)

44 PBSCT.tw. (2851)

45 ((autologous or auto-logous or auto) adj (PBSC or PSC)).tw. (467)

46 (autoPBSCT or autoPBSC or autoPSC).tw. (97)

47 CBSCT.tw. (69)

48 Hematopoietic Stem Cell Mobilization/ (8427)

49 ((hematopoietic or hemato-poietic or HSC or HSCs) adj10 mobili#ation).tw. (2457)

50 Transplantation, Autologous/ (70268)

51 (transplant* adj1 (autologous or auto-logous)).tw. (8071)

52 (autograft* or auto-graft* or autotransplant* or auto-transplant*).tw. (41108)

53 exp Transplantation, Homologous/ (110758)

54 (transplant* adj1 (homologous or homo-logous or allogeneic or allo-geneic or isogeneic or iso-geneic or syngeneic)).tw. (11199)

55 (allotransplant* or allo-transplant* or allograft* or allo-graft* or homograft* or homo-graft* or isograft* or iso-graft*).tw. (143292)

56 Bone Marrow Transplantation/ (85051)

57 (bone marrow adj2 (graft* or transplant*)).tw. (70135)

58 exp "Cell- and Tissue-Based Therapy"/ (1189439)

59 ((cell or cells or cell-based or tissue$1 or tissue-based) adj (therapy or therapies or transplant*)).tw. (129614)

60 or/9-59 (2394286)

61 8 and 60 (5910)

62 exp animal experimentation/ or exp models, animal/ or animals/ or mammals/ or vertebrates/ or exp fishes/ or exp amphibia/ or exp reptiles/ or exp birds/ or exp hyraxes/ or exp marsupialia/ or exp monotremata/ or exp scandentia/ or exp chiroptera/ or exp carnivora/ or exp cetacea/ or exp Xenarthra/ or exp elephants/ or exp insectivora/ or exp lagomorpha/ or exp rodentia/ or exp sirenia/ or exp Perissodactyla/ or primates/ or exp strepsirhini/ or haplorhini/ or exp tarsii/ or exp platyrrhini/ or catarrhini/ or exp cercopithecidae/ or gorilla gorilla/ or pan paniscus/ or pan troglodytes/ or exp pongo/ or exp hylobatidae/ or hominidae/ (10738734)

63 (animal$1 or chordata or vertebrate* or fish$2 or amphibian* or amphibium* or reptile$1 or bird$1 or mammal* or dog or dogs or canine$1 or cat or cats or hyrax* or marsupial* or monotrem* or scandentia or bat or bats or carnivor* or cetacea or edentata* or elephant* or insect or insects or insectivore or lagomorph* or rodent$2 or mouse or mice or murine or murinae or muridae or rat or rats or pig or pigs or piglet$1 or swine or rabbit$1 or sheep$1 or goat$1 or horse$1 or equus or cow or cows or cattle or calf or calves or bovine or sirenia or ungulate$1 or primate$1 or prosimian* or haplorhini* or tarsiiform* or simian*or platyrrhini or catarrhini or cercopithecidae or ape or apes or hylobatidae or hominid* or chimpanzee* or gorilla* or orangutan* or monkey or monkeys or ape or apes).tw. (8554114)

64 exp Drug Evaluation, Preclinical/ (306518)

65 (preclinic* or pre-clinic*).tw. (140341)

66 or/62-65 (12502571)

67 61 and 66 (1789)

68 67 use prmz (540)

69 exp pulmonary hypertension/ (88479)

70 (pulmonary adj2 hypertens*).tw. (76144)

71 (PAH).tw. (36107)

72 or/69-71 (132392)

73 monocrotaline/ (3008)

74 (monocrotalin* or MCT).tw. (10828)

75 73 or 74 (11229)

76 72 or 75 (140985)

77 exp stem cell/ (338939)

78 ((stem or progenitor* or mother or precursor*) adj1 cell$1).tw. (455508)

79 exp bone marrow cell/ (237902)

80 (bone marrow adj3 cell$1).tw. (122869)

81 (BMD-EPC or BMD-EPCs or BMDEPC or BMDEPCs).tw. (23)

82 ("colony-forming" adj (cell$1 or unit or units)).tw. (38308)

83 (CFU or CFUs).tw. (71887)

84 ((embryoid or embroid or embryonic) adj2 (body or bodies or cell$1)).tw. (66276)

85 (angioblast* or hemangioblast* or haemangioblast*).tw. (6922)

86 myoblast$1.tw. (22839)

87 ((haemogenic or hemogenic) adj2 (endothelial or endothelium)).tw. (311)

88 ((osteoprogenitor adj cell$1) or OPC or OPCs).tw. (9125)

89 (side adj population adj cell$1).tw. (842)

90 (circulating angiogenic adj (cell or cells)).tw. (299)

91 (CAC or CACs).tw. (8858)

92 IPS cell$1.tw. (3869)

93 hiPSC$1.tw. (1124)

94 common lymphoid progenitor$1.tw. (516)

95 (("Pre-B" or "Pro-B") adj1 (cell$1 or lymphocyte*)).tw. (8285)

96 (("B-cell" or "B-cells" or B-lymphcyte$1 or B-lymphoid$1) adj1 (immatur* or precursor* or progenitor* or transitional)).tw. (8221)

97 (("T-cell" or "T-cells" or T-lymphcyte$1 or T-lymphoid$1) adj1 (immatur* or precursor* or progenitor* or transitional)).tw. (5507)

98 (thymocyte* or T-lymphocyte*).tw. (209185)

99 ((mesenchymal adj3 (stem or stroma$1 or progenitor* or precursor*)) and cell$1).tw. (55069)

100 (MSC or MSCs or ADMSC or ADMSCs or BM-MSC or BM-MSCs or BMD-MSC or BMD-MSCs or BMDMSC or BMDMSCs).tw. (35017)

101 ((multipotent or multi-potent) adj3 (stroma$1 cell$1 or stem cell$1)).tw. (6514)

102 marrow stroma$1 cell$1.tw. (12298)

103 CFU-F$1.tw. (1384)

104 mesenchymal.tw. and exp Gene Therapy/ (1740)

105 exp stem cell transplantation/ (140672)

106 ((hematopoietic or hemato-poietic or HSC or HSCs) adj10 transplant*).tw. (45247)

107 ((autologous or auto-logous or auto) adj HCT).tw. (534)

108 autoHCT.tw. (61)

109 ((allogeneic or allo-geneic or homologous or homo-logous) adj HCT).tw. (1436)

110 ((PBSC or PBSCs) adj10 transplant*).tw. (2248)

111 PBSCT.tw. (2851)

112 ((autologous or auto-logous or auto) adj (PBSC or PSC)).tw. (467)

113 (autoPBSCT or autoPBSC or autoPSC).tw. (97)

114 CBSCT.tw. (69)

115 stem cell mobilization/ (8427)

116 ((hematopoietic or hemato-poietic or HSC or HSCs) adj10 mobili#ation).tw. (2457)

117 autotransplantation/ (26878)

118 (transplant* adj1 (autologous or auto-logous)).tw. (8071)

119 allotransplantation/ (32679)

120 (transplant* adj1 (homologous or homo-logous or allogeneic or allo-geneic or isogeneic or iso-geneic or syngeneic)).tw. (11199)

121 (allotransplant* or allo-transplant* or allograft* or allo-graft* or homograft* or homo-graft* or isograft* or iso-graft*).tw. (143292)

122 exp bone marrow transplantation/ (97871)

123 (bone marrow adj2 (graft* or transplant*)).tw. (70135)

124 ((cell or cells or cell-based or tissue$1 or tissue-based) adj (therapy or therapies or transplant*)).tw. (129614)

125 or/77-124 (1349117)

126 76 and 125 (3044)

127 exp animal experiment/ or exp animal model/ or animal/ or exp invertebrate Chordata/ or exp experimental animal/ or exp transgenic animal/ or exp male animal/ or exp female animal/ or exp juvenile animal/ or vertebrate/ or exp fish/ or exp amphibia/ or exp reptile/ or exp bird/ or mammal/ or exp hyrax/ or exp marsupial/ or exp monotremate/ or exp scandentia/ or placental mammals/ or exp bat/ or exp carnivora/ or exp cetacea/ or exp edentata/ or exp elephant/ or exp insectivora/ or exp lagomorph/ or exp rodent/ or exp sirenia/ or exp ungulate/ or primate/ or exp prosimian/ or haplorhini/ or exp tarsiiform/ or simian/ or exp platyrrhini/ or catarrhini/ or exp cercopithecidae/ or ape/ or exp hylobatidae/ or hominid/ or exp chimpanzee/ or exp gorilla/ or exp orang utan/ (11140546)

128 (animal$1 or chordata or vertebrate* or fish$2 or amphibian* or amphibium* or reptile$1 or bird$1 or mammal* or dog or dogs or canine$1 or cat or cats or hyrax* or marsupial* or monotrem* or scandentia or bat or bats or carnivor* or cetacea or edentata* or elephant* or insect or insects or insectivore or lagomorph* or rodent$2 or mouse or mice or murine or murinae or muridae or rat or rats or pig or pigs or piglet$1 or swine or rabbit$1 or sheep$1 or goat$1 or horse$1 or equus or cow or cows or cattle or calf or calves or bovine or sirenia or ungulate$1 or primate$1 or prosimian* or haplorhini* or tarsiiform* or simian*or platyrrhini or catarrhini or cercopithecidae or ape or apes or hylobatidae or hominid* or chimpanzee* or gorilla* or orangutan* or monkey or monkeys or ape or apes).tw. (8554114)

129 (preclinic* or pre-clinic*).tw. (140341)

130 or/127-129 (12706717)

131 126 and 130 (1252)

132 131 use emczd (763)

133 68 or 132 (1303)

134 remove duplicates from 133 (925) [total unique records]

135 134 use prmz (514) [unique MEDLINE records]

136 134 use emczd (411) [unique Embase records]
